# Urine protein biomarkers of bladder cancer arising from 16-plex antibody-based screens

**DOI:** 10.18632/oncotarget.27941

**Published:** 2021-04-13

**Authors:** Kamala Vanarsa, Shereen Enan, Pooja Patel, Briony Strachan, Anto Sam Crosslee Louis Sam Titus, Aphrihl Dennis, Yair Lotan, Chandra Mohan

**Affiliations:** ^1^Department Biomedical Engineering, University of Houston, Houston, TX, USA; ^2^Department of Urology, UT Southwestern Medical Center, Dallas, TX, USA

**Keywords:** urothelial, proteomics, targeted screens, interleukins, inflammation

## Abstract

Purpose: The purpose of this study is to identify novel urine protein biomarkers of bladder cancer using a Luminex based screening platform.

Materials and Methods: The current study examines urine samples from 66 subjects, comprised of 31 Urology clinic controls and 35 bladder cancer patients, using a Luminex based screening platform. ELISA validation was carried out for the top 4 prospective urine biomarkers using an independent cohort of 20 Urology clinic controls and 60 bladder cancer (BC) subjects.

Results: Of the 16 proteins screened by Luminex, 10 showed significant elevation in BC compared to the controls. Eight of these urine proteins were able to differentiate BC from control urine with ROC AUC values exceeding 0.70 at *p* < 0.0001, with specificity values exceeding 0.9. Upon ELISA validation, urine IL-1α, IL-1ra, and IL-8 were able to distinguish control urine from urine drawn from various bladder cancer stages, with IL-8 being the best discriminator. Compared to members of the IL-1 cytokine family, urine IL-8 was also best at discriminating T1 and/or T2–T4 from Ta BC (ROC AUC ≥ 0.83), as well as high grade from low grade BC (ROC AUC ≥ 0.82).

Conclusions: These findings suggest that urine IL-1α, IL-1ra and IL-8 are useful indicators of bladder cancer. Urine IL-8 not only distinguishes bladder cancer from controls, it also discriminates high grade from low grade disease, and the successive clinical stages of bladder cancer. While supportive of previous reports, these findings warrant further analysis in prospective cohorts.

## INTRODUCTION

Bladder cancer (BC) is the sixth most common cancer diagnosis in the United States and is over four times more common in men than women [1, 2]. In terms of demographics, Whites are more likely to be diagnosed with bladder cancer than African Americans or Hispanic Americans and the disease incidence increases with age [3]. The most common diagnostic methods for BC include cytology and cystoscopy. Studies continue to examine potential BC biomarkers in urine samples as an alternative method of detection [4, 5].

Urine cystoscopy is currently the “gold standard” for diagnosis of BC. However, it is relatively invasive, expensive, and can potentially cause urinary tract infections. Other possible complications include hematuria, dysuria, and injury to the bladder or urethra. Sensitivity and specificity range from 62–84% and 43–98%, respectively [6]. Urine cytology is a non-invasive method most commonly used for the surveillance of BC, but it is not recommended in initial disease evaluation [7]. Although it exhibits high specificity for high-grade tumors (~86%), it has poor sensitivity for low-grade tumors (~16%) (2). For BC patients presenting with low-grade tumors with high rates of recurrence, this diagnostic modality may not be reliable for diagnosis [8].

In contrast, urine is a noninvasive and readily available biological fluid that can be used for diagnostic tests. Urine biomarkers could potentially provide preliminary confirmation of low-grade BC before invasive procedures are performed and facilitate surveillance of BC, as reviewed [9]. Finally, urine can be collected and even tested serially by the patient, using various cost-effective point-of-care diagnostic tools compared to other methods of detection [10].

Most previous studies have utilized ELISA based assays for monitoring a limited number of urine proteins in bladder cancer, as reviewed [4]. The present study implements a Luminex based screening platform with a cytokine/chemokine panel that simultaneously interrogates 16 urine biomarkers, followed by ELISA validation of 4 prospective urine biomarkers. Luminex screening is advantageous over ELISA as it allows for the quantitative analysis of multiple biomarkers simultaneously, rather than the measurement of a single protein at a time [11, 12]. The magnetic beads used in the Luminex system have a large surface area, increasing the range of detection and respond rapidly and efficiently to a magnetic field. The simultaneous screening of multiple biomarkers provides increased confidence in the detection and classification of bladder cancer.

Specifically, the 16 urine proteins interrogated include Eotaxin, Groα, IFNα, IL-1α, IL-1ra, IL-7, IL-8, IL-15, IL-31, IP-10, MIP-1α, MIP-1β, MCP-1, RANTES, SDF-1α, and TNFβ. Most of these proteins are inflammatory cytokines or chemokines that are important for chemoattraction of various leukocytes into target tissue. Apart from IL-8 and IL-1ra, the other candidates interrogated have not been reported previously as urinary biomarkers for BC. These specific molecules were interrogated in this study since yet other cytokines/chemokines have been implicated previously in BC pathogenesis.

The current study examines urine samples from 66 subjects (31 urology clinic controls and 35 BC). Of the 16 proteins screened by Luminex, 12 were within the detectable range and among these, 10 urine biomarkers showed significant elevation in BC compared to the controls. Of these 7 proteins were able to discriminate BC from control urine at ROC AUC values exceeding 0.7 (*p* < 0.0002). Four of these 7 proteins were selected for further ELISA validation because they belonged to different correlation clusters. ELISA validation for these 4 urine biomarkers was carried out using an independent cohort of 20 urology clinic controls and 60 BC subjects. Of these 4 proteins, IL-8 displayed the highest significance in discriminating between controls and BC patients and discriminating highly advanced stages/grades of BC from less advanced stages/grades of BC.

## RESULTS

### Luminex based screening

**Figure 1:**
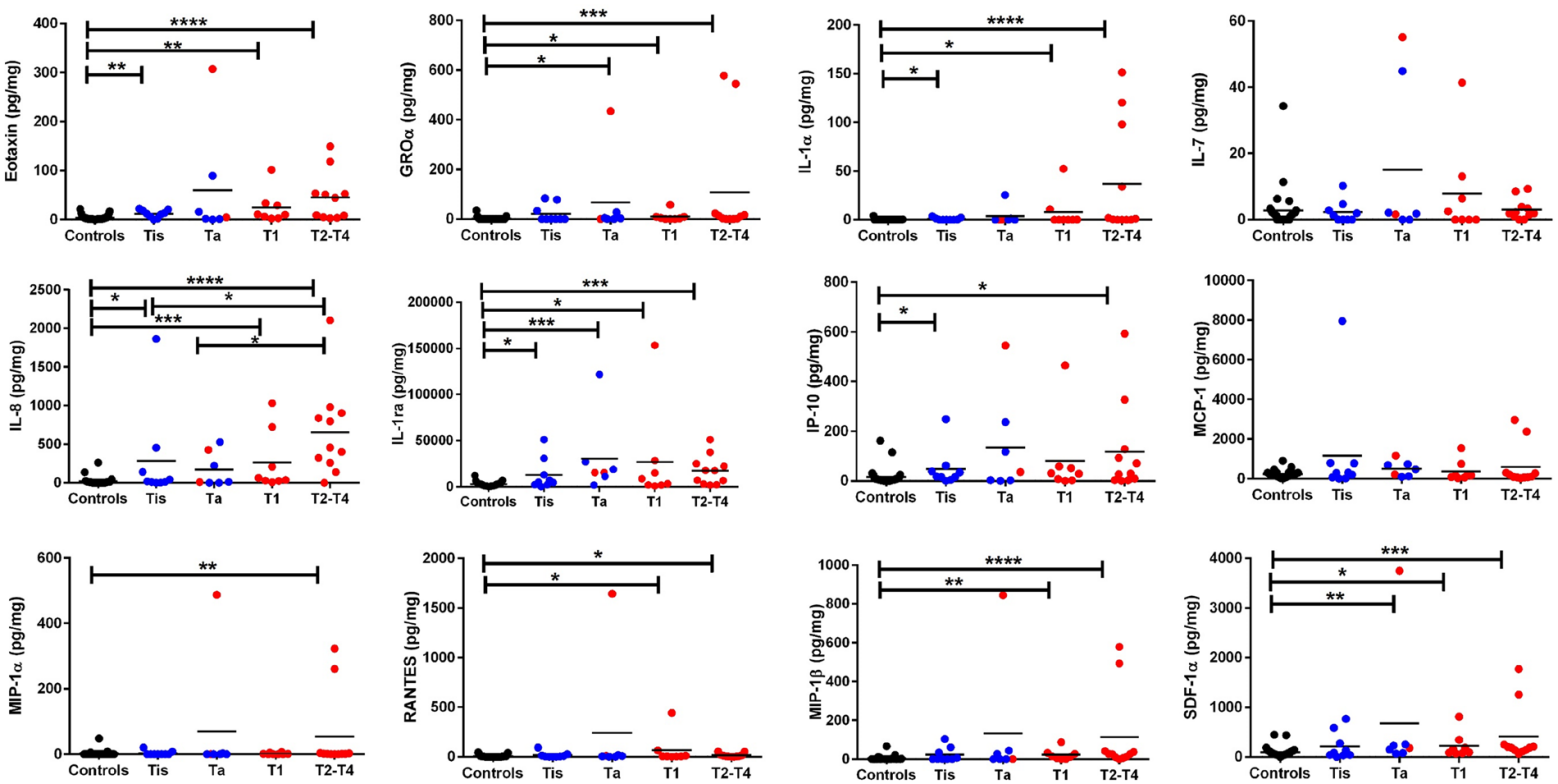
Luminex based screen of 16 urine proteins in bladder cancer. The dot plots depict the 12 proteins that were detectable by Luminex assay either in BC urine or the urology clinic controls, labeled as “Controls”. Tested samples included 31 controls, 7 Ta, 9 Tis, 8 T1, and 11 T2–T4 urine samples. Creatinine normalized urine protein levels are shown in different colors specific for each group (black dots = controls, blue dots = low grade BC, and red dots = high grade BC). Low grade tumor and high grade tumor classification was based on pathology reports. The asterisks designate the level of significance between the different groups: ^*^ = *p* < 0.05, ^**^ = *p* < 0.01, ^***^ = *p* < 0.001, and ^****^ = *p* < 0.0001, using a Mann Whitney *U* test. The primary data for this analysis is presented in Supplementary Table 2. All controls used for this study were drawn from the Urology clinic, including patients investigated for hematuria but found not to have any urological cancers.

**Figure 2:**
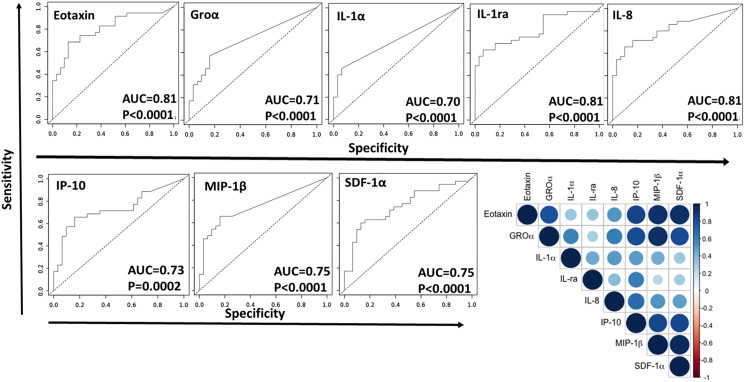
Eight urine proteins that discriminate BC from controls based on the Luminex-based screen of 16 proteins. Receiver Operating Curve Area Under Curve (ROC-AUC) plots were generated for eight urine proteins including Eotaxin, GROα, IL-1α, IL-1ra, IL-8, IP-10, MIP-1β, and SDF-1α to determine their ability to discriminate BC from controls. AUC values and *p*-values are listed on each curve. The closer the AUC value is to 1, the higher the discriminatory potential of the protein to distinguish between the two groups, with maximized specificity and sensitivity. All of the proteins exhibited AUC values of 0.70 or higher, with *p*-values < 0.0001, except IP-10 which had a *p*-value of 0.0002. A correlation plot was also generated for these eight urine proteins. Each circle represents the degree of correlation for the given protein pair, with blue intensity corresponding to positive correlation and red intensity corresponding to negative correlation.

Urine samples from 60 male and 6 female subjects (controls = 31, Ta = 7, Tis = 9, T1 = 8, and T2–T4 = 11), age 43–86 years, were used for the Luminex screening. Of the 16 potential cytokine biomarkers assayed, only 12 biomarkers were within the detectable range. Among them, 10 biomarkers showed a significant increase in BC compared to the urology clinic controls (Figure 1). The urine proteins that were significantly elevated in at least one of the BC groups, include Eotaxin, Groα, IL-8, IL-1α, IL-1ra, IP-10, MIP-1β, MIP-1α, RANTES and SDF-1a. Eight of these urine proteins were able to differentiate BC from control urine with ROC AUC values exceeding 0.70 at *p* < 0.0001, with specificity values exceeding 0.9 (Figure 2, Supplementary Table 2). Several of these urine proteins were highly correlated with each other, as displayed in the correlation plot in Figure 2. IL-1α, IL-1ra, IL-8 and SDF-1α were selected for validation by ELISA in an independent sample cohort as they were significantly elevated in BC urine, and not as highly correlated with each other.

### ELISA validation of Hits from the Luminex screen

**Figure 3:**
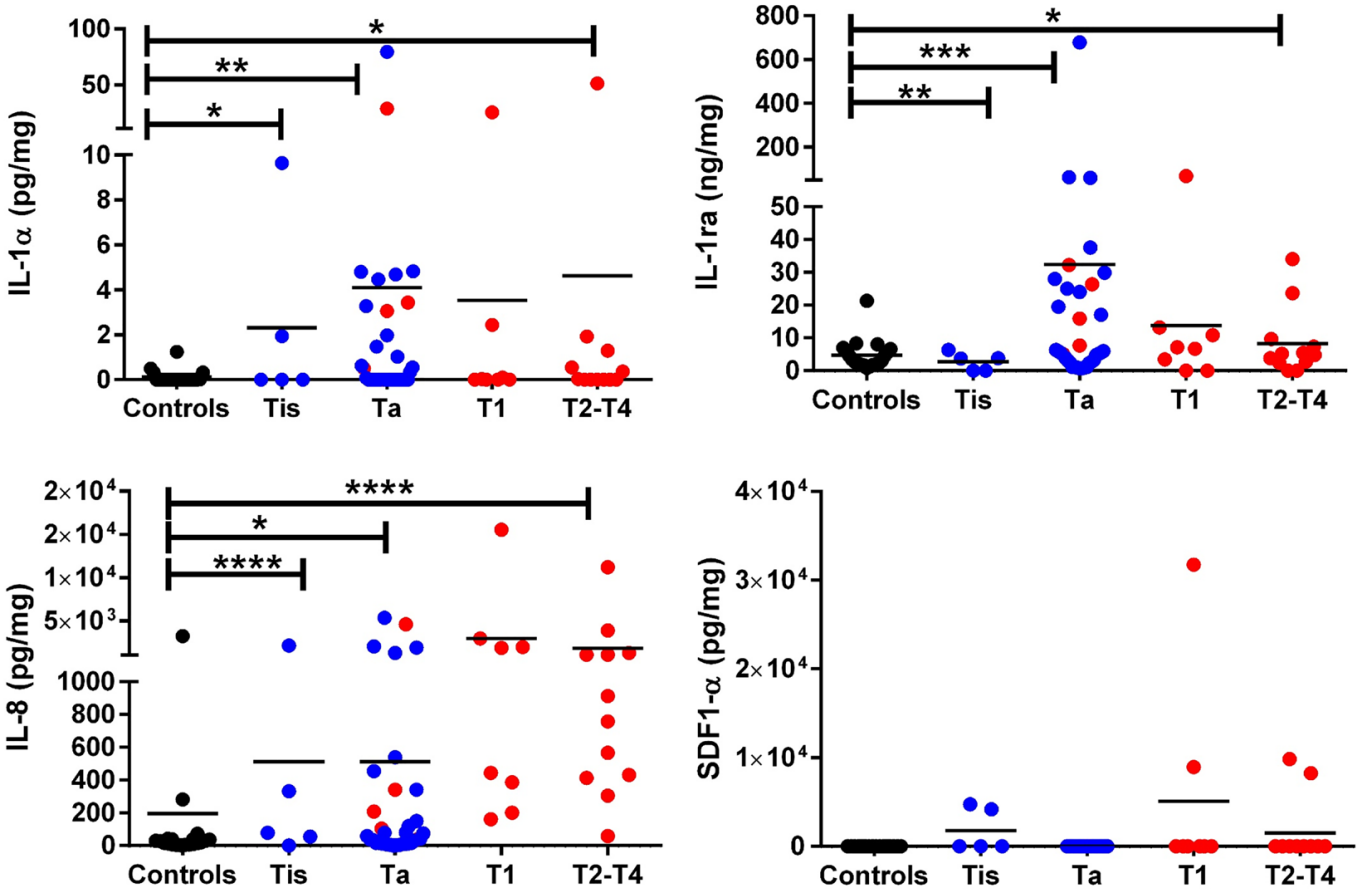
ELISA validation of IL-1α, IL-1ra, IL-8, and SDF-1α in bladder cancer patients with varying clinical stages. The dot plots depict the expression of IL-1α, IL-1ra, IL-8, and SDF-1α in urine from different stages of BC. Included were 20 urology clinic controls (“controls”), 35 Ta, 5 Tis, 8 T1, and 12 T2-T4 BC patients. Creatinine normalized urine protein levels are shown in different colors (black dots = controls, blue dots = low grade BC and red dots = high grade BC). Low grade tumor and high grade tumor classification was based on pathology reports. The asterisks designate the level of significance between the different groups: ^*^= *p* < 0.05, ^**^= *p* < 0.01, ^***^= *p* < 0.001, and ^****^ = *p* < 0.0001, using a Mann Whitney *U* test.

An independent cohort of 80 urine samples (20 urology clinic controls, 35 Ta, 5 Tis, 8 T1, and 12 T2-T4) was used for ELISA validation of IL-1α, IL-1ra, IL-8, and SDF-1α. The latter group included 8 patients with T2, 2 with T3 and 2 with T4 BC. The mean and median values of each urine protein biomarker in all sample groups, and the fold change, AUC, sensitivity, specificity, NPV, PPV values are summarized in Supplementary Table 1. Once again, IL-1α, IL-1ra, and IL-8 were significantly elevated in BC urine compared to the controls; however, SDF-1a failed to distinguish these groups (Supplementary Figure 1). Urine IL-1α, IL-1ra, and IL-8 were able to distinguish urine from controls and Ta as well as urine from controls and T2–T4, with IL-8 being the best discriminator (Figure 3 and Supplementary Table 1). Urine IL-8 and IL-1ra were also able to distinguish T1 BC from the controls, again with urine IL-8 being the better discriminator (Figure 3 and Supplementary Table 1).

**Figure 4:**
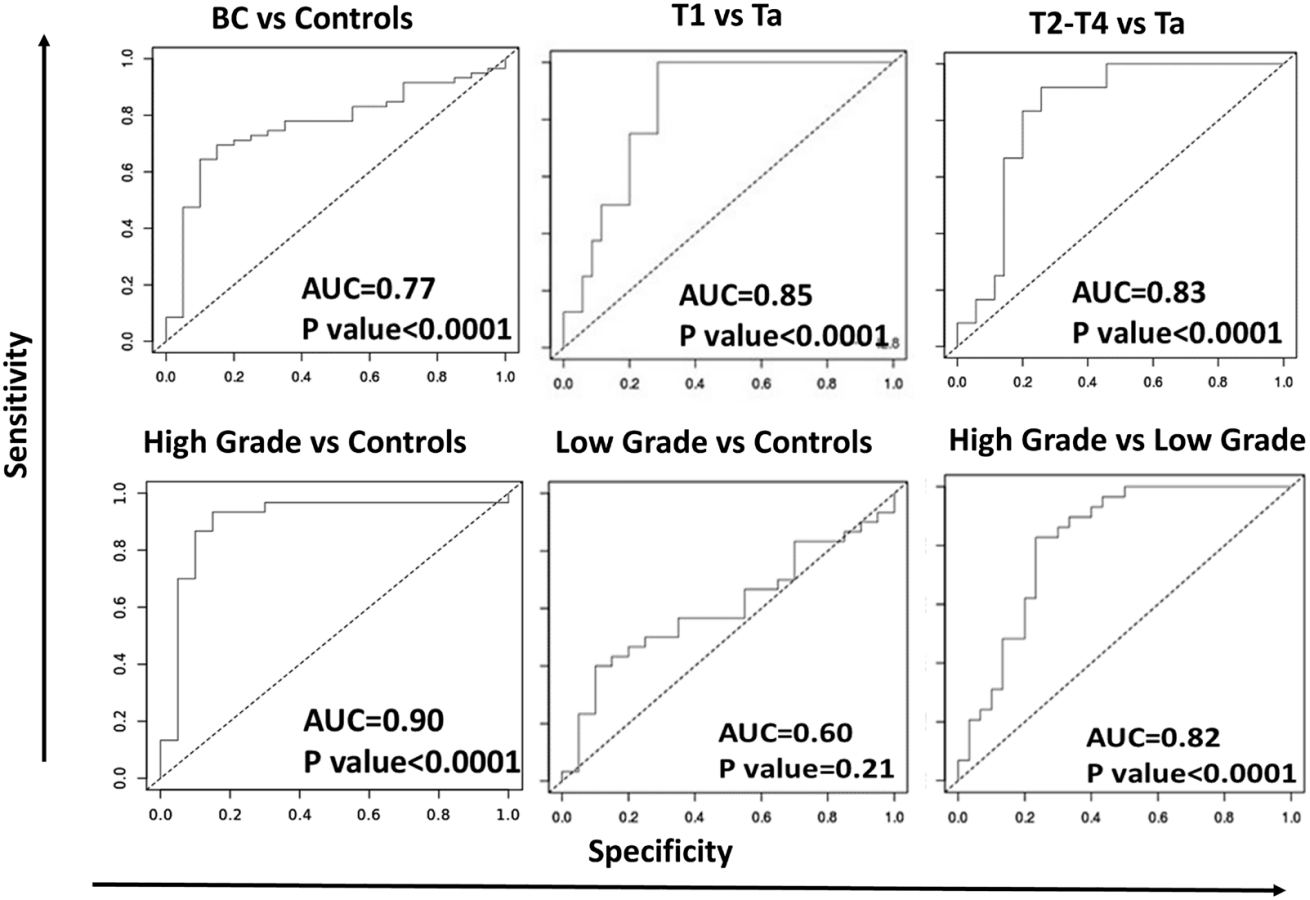
ROC-AUC curves for urine IL-8 in distinguishing different stages of bladder cancer. ROC-AUC curves were generated for urine IL-8 to determine its discriminatory capability among different BC groups. AUC values and *p*-values are listed on each curve. The closer the AUC value is to 1, the higher the discriminatory potential of the protein to distinguish between the two sample groups, with maximized sensitivity and specificity. All comparisons exhibited AUC values of 0.77 or higher, with *p*-values < 0.0001, except for the comparison between low grade versus urology clinic controls.

Besides comparing the different BC groups to the controls, the more advanced BC stages were also compared to the less advanced stages, using ROC (Figure 4). Of the ELISA-tested proteins, urine IL-8 was best at discriminating T1 and/or T2-T4 from Ta BC (ROC AUC ≥ 0.83), as well as high grade BC from low grade BC (ROC AUC ≥ 0.82) (Figure 4).

## DISCUSSION

Methods used in this study utilize highly specific antibody-protein interactions to attain quantitative accuracy for both low and high abundance proteins. In this study, Luminex screening was used to simultaneously assay the protein abundances of 16 potential biomarkers in different stages of bladder cancer and then compared to urology clinic controls. Of the 16 proteins screened by Luminex, 12 were in the detectable range in the assayed samples among which 10 urine proteins showed significant differences between the urology controls and different stages of bladder cancer. ELISA validation was performed on 4 prospective urine biomarkers; of these IL-1α, IL-1ra, and IL-8 were validated to be significantly elevated in BC, with urine IL-8 demonstrating the best ability to distinguish more advanced stages and grades of BC from the less advanced stages and grades of BC.

IL-1α, a cytokine of the IL-1 family, plays a vital role in both immunity and inflammation [13]. The finding that urine IL-1α is elevated in BC is relatively novel since only one other study has noted that urine IL-1α may be induced following intravesical immunotherapy with BCG in noninvasive bladder cancer [14]. In the present study, IL-1α was noted to be elevated not only in BC compared to controls, but also in the more advanced stages of BC, and in higher grades of BC, compared to the less advanced stages/grades, based on data from 2 orthogonal platforms, Luminex and ELISA. On both platforms, urine IL-1α also exhibited some of the highest PPV and specificity values, mainly because the controls exhibited consistently low levels of urine IL-1α.

IL-1 receptor antagonist (IL-1ra) is an antagonist in the IL-1 family of cytokines [15]. Previous studies have shown that specific IL-ra gene polymorphisms are associated with BC [16]. Another study has demonstrated that serum levels of IL-1ra are significantly higher in BC patients as compared to controls [17]. Recently, Kamat et al. have reported that urine IL-1ra may be used to identify the risk of recurrence in BC patients [18]. Our findings are consistent with earlier reports, in that urine IL-1ra was significantly elevated in BC patients, from the Ta stage onwards, based on data from 2 orthogonal platforms, Luminex and ELISA, with high PPV and specificity values, mainly because the controls exhibited consistently low levels of urine IL-1ra.

IL-8, a cytokine in the IL-1 family, plays an important role in immunity against pathogens [19]. IL-8 has been studied in bladder tumor biology and previous studies indicate that elevated levels of urinary IL-8 may be informative in BC diagnosis [20, 21]. IL-8 levels are significantly higher in BC patients in comparison to the controls [17, 20]. In other studies, IL-8 emerged as a promising biomarker for predicting the likelihood of BC recurrence [18, 22, 23]. A previous study showed that IL-8 was present in higher concentrations in high grade bladder cancer urine [17]. Our findings confirmed these earlier reports as IL-8 proved to best discriminate between BC and controls, but also discriminate high grade BC, low grade BC, Ta to T1 and T2 and more severe BC groups. This study also showed that based on data from 2 orthogonal platforms, Luminex and ELISA, IL-8 had high PPV and specificity values.

SDF-1 is a ligand for the chemokine receptors CXCR4 and CXCR7 and induces proliferation of bladder cancer cells via the activation of CXCR4 [24]. SDF-1a levels are elevated in bladder cancer patients with metastasis compared to those without metastasis and increased levels of SDF-1 were correlated with increased depth of invasion [25, 26]. In our study, both the urology controls and Ta groups had very low levels of SDF-1 (below detection limits) however Tis, T1 and T2 groups had highly elevated SDF-1 which corroborates previous studies (Supplementary Table 1). It is possible that more sensitive diagnostic platforms may be able to better detect the relatively low levels of SDF1 in BC urine (compared to other more prevalent markers).

Besides the 4 proteins selected for ELISA validation, the initial Luminex screen also identified urine Eotaxin, GROα, IP-10 and MIP-1β as having the capacity to distinguish BC from controls, with ROC AUC values exceeding 0.70 at *p* < 0.0001, with specificity values exceeding 0.9. Importantly, all 4 appear to be novel as they have not been pursued before as BC markers. Eotaxin is a member of the CC chemokine family and plays a major role in immunoregulatory processes [27]. Growth-regulated protein alpha (GROα) is a chemokine involved in both inflammation and tumor development [28]. Interferon-inducible protein 10 (IP-10) regulates inflammation and mediates chemotaxis of many cells types [29]. Macrophage inflammatory protein beta (MIP-1β) activates cells of the immune system and functions in the synthesis of pro-inflammatory cytokines [30]. Clearly validation studies are warranted to delineate the potential roles of these chemokines in BC diagnostics.

These studies indicate that urine IL-1α, IL-1ra, and IL-8 are potential biomarkers of BC, two of which re-affirm previous reports. These studies shed additional light on the potential utility of these markers, since some of them (e.g., urine IL-8) also exhibit the ability to discriminate T1 and/or T2-T4 from Ta BC, as well as high grade from low grade BC. Looking forward, systematic studies in larger patient cohorts are warranted to establish the specific clinical contexts in which these markers may be used, including the following: (i) for initial diagnosis of BC, (ii) for surveillance of tumor recurrence, and/or (ii) for assessing treatment response following BCG therapy or other therapeutic modalities. Finally, these newer urine biomarkers need to be compared against the performance of current yardsticks such as the Bladderchek and UroVysion FISH assay.

## MATERIALS AND METHODS

### Patients and sample collection

BC urine samples and Urology clinic controls were obtained with informed patient consent from the University of Texas Southwestern Medical Center (UTSW) in Dallas, Texas. The Urology clinic controls included patients investigated for hematuria, but found not to have any urological cancers. The study was approved by the institutional review boards at the University of Houston, Houston, Texas and UTSW, Dallas, Texas. Urine samples were centrifuged, aliquoted and stored at –80°C. For Luminex based proteomic screening, a total of 66 samples were utilized, comprised of 31 urology clinic controls and 35 bladder cancer (BC) urine samples, including, 7 Ta (noninvasive papillary carcinoma), 9 Tis (flat carcinoma *in situ*), 8 T1 (tumor spread to connective tissue), and 11 patients with more advanced cancer (muscle-invasive bladder cancer), including 5 with T2, 3 with T3 and 3 with T4x BC. Among the BC patients, 10 had diabetes (on anti-diabetic medications), 18 had hypertension (on anti-hypertensive medications) and 14 had various cardiovascular diseases (and were on statins, anti-coagulants, diuretics or other cardiovascular medications). Patient demographics are detailed in Table 1.

**Table 1:** Demographics of subjects used for bladder cancer urine biomarker studies

		Subjects Used for Luminex Based Screen of Urine Biomarkers				
Variable	Category	Controls (*N* = 31)	Ta (*N* = 7)	Tis (*N* = 9)	T1 (*N* = 8)	T2–T4 (*N* = 11)
Age (years)		65.93 ± 9.77	70.29 ± 9.82	71.00 ± 6.06	68.75 ± 7.24	74.45 ± 7.60
Gender, *n* (%)	Male	28 (90.32%)	5 (71.43%)	9 (100.00%)	7 (87.50%)	11 (100.00%)
	Female	3 (9.68%)	2 (28.57%)	0 (0.00%)	1 (12.50%)	0 (0.00%)
Race; *n* (%)	African American	0 (0.00%)	0 (0.00%)	0 (0.00%)	0 (0.00%)	0 (0.00%)
	Asian	0 (0.00%)	0 (0.00%)	0 (0.00%)	0 (0.00%)	0 (0.00%)
	Caucasian	31 (100.00%)	7 (100.00%)	9 (100.00%)	8 (100.00%)	11 (100.00%)
	Hispanic	0 (0.00%)	0 (0.00%)	0 (0.00%)	0 (0.00%)	0 (0.00%)
		**Subjects Used for ELISA Validation of Urine Biomarkers**				
		**Controls (*N* = 20)**	**Ta (*N* = 35)**	**Tis (*N* = 5)**	**T1 (*N* = 8)**	**T2–T4 (*N* = 12)**
Age (years)		68.5 ± 11.4	67.6 ± 12.2	68.8 ± 3.7	74.375 ± 6.1	71.9 ± 11.2
Gender, *n* (%)	Male	17 (85%)	26 (74.3%)	5 (100%)	5 (62.5%)	11 (91.6%)
	Female	3 (15%)	9 (25.7%)	0 (0.0%)	3 (37.5%)	1 (8.33%)
Race, *n* (%)	African American	3 (15%)	2 (5.7%)	0 (0.0%)	0 (0.0%)	0 (0.0%)
	Hispanic	1 (5%)	3 (8.6%)	0 (0.0%)	0 (0.0%)	0 (0.0%)
	Latin America	1 (5%)	0 (0.0%)	0 (0.0%)	0 (0.0%)	0 (0.0%)
	Caucasian	14 (70%)	30 (85.7%)	5 (100%)	8 (100%)	12 (100%)
	Other	1 (5%)	0 (0.0%)	0 (0.0%)	0 (0.0%)	0 (0.0%)

Data are presented as *n* (%) or average ± SD as appropriate. The T2–T4 group included 8 patients with T2, 2 with T3 and 2 with T4 BC. The controls comprised of urology clinic controls, without any urological cancers.

For the subsequent ELISA validation study, a total of 80 samples were utilized, comprised of 20 urology clinic controls and 60 BC urine samples, including, 35 Ta, 5 Tis, 8 T1, and 12 patients with more advanced cancer (muscle-invasive bladder cancer), including 8 with T2, 2 with T3 and 2 with T4. Among these patients, 12 had diabetes (on anti-diabetic medications), 28 had hypertension (on anti-hypertensive medications) and 18 had various cardiovascular diseases (and were on statins, anti-coagulants, diuretics or other cardiovascular medications). Patient demographics are detailed in Table 1. Of these patients, 36 had trans-urethral resection of bladder tumor (TURBT), 11 had cystectomy, 3 had cystoprostatectomy, and the rest only had a biopsy. All patients who underwent TURBT were clinical node negative and non-metastatic. Of the 11 patients who underwent cystectomy, 6 were staged as T2 N0 Mx, 1 as T2b N2 Mx, 1 as T3b N3 Mx, 1 as T4a N1 Mx, 1 as T4a N2 Mx, and 1 as high grade T1. Of the 35 patients with Ta BC, 30 had low grade tumor while the rest had high grade tumor, based on pathology reports.

For both the screening and validation cohorts, the inclusion criteria for BC was biopsy proven BC, while exclusion criteria excluded patients on dialysis, patients on systemic chemotherapy, patients with active urinary tract infections, symptomatic stone disease, other non-urothelial active cancers and patients who could not void (so no samples were drawn from catheters). All BC urine samples were prospectively collected from newly diagnosed patients, before surgery. All controls used in both the screening and validation cohorts were subjects being investigated in the Urology clinic for BC, but found to be negative for BC (or other malignancies).

### Luminex based protein screen

Luminex micro-bead assay (Cat #: HCYTMAG-60K-PX41, Lot #: 3090739) uses conjugated-microsphere particles to capture specific antigens onto their surface. Through two fluorescence signals, the instrument determines the analyte from the bead and its corresponding concentration from the intensity of the detection dye. Thus, the Luminex system allows for the detection and quantification of multiple biomarkers simultaneously. This assay was used to screen 16 potential urine biomarkers (including Eotaxin, Groα, IFNα, IL-1α, IL-1ra, IL-7, IL-8, IL-15, IL-31, IP-10, MIP-1α, MIP-1β, MCP-1, RANTES, SDF-1α, and TNFβ) to determine their expression levels in BC urine samples when compared to the control urine samples. Standards or urine samples diluted 1:50 and beads were added to the wells. After incubation, the plate was washed before biotinylated detection antibodies were added. Following incubation, Streptavidin-Phycoerythrin (detection dye) was added, incubated, and then washed. Finally, sheath fluid was added into each well, and the plate was read, collecting 50 beads per analyte.

### ELISA

Prospective biomarkers selected from the Luminex screen were validated by ELISA. Human SDF-1α ELISA kit (catalog#ELH-SDF-1α), Human IL-8 ELISA kit (catalog #ELH-IL-8), Human IL-1ra ELISA kit (catalog# ELH-1L-1ra), and Human IL-1α ELISA kit (catalog#ELH-IL-1α) were purchased from Ray Biotech, GA and used following manufacturer protocol. Urine samples were diluted 1 in 20 for IL-8 and IL-1ra and 1 in 2 for IL1-α and SDF-1α. Levels of urine creatinine were assayed using the Creatinine Parameter Assay Kit (catalog#KEG005, R&D systems). The absolute levels of urine protein biomarkers were determined using standard curves run on each ELISA plate, and normalized by urine creatinine concentration.

### Statistical analysis

All data collected was plotted and analyzed using Graph Pad prism 7, Microsoft Excel, and R studio. Statistical differences between the sample groups were determined using Mann Whitney *U* test and chi square test. Sensitivity, specificity, area under the curve (AUC), predictive positive values (PPV), and negative predictive values (NPV) were calculated using the easyROC software.

## SUPPLEMENTARY MATERIALS






