# Supplementary material for: Urine protein biomarkers of bladder cancer arising from 16-plex antibody-based screens
Source: Oncotarget. 2021 Apr 13;12(8):783–90. doi: 10.18632/oncotarget.27941 (PMC8057279; doi:10.18632/oncotarget.27941)
Supplement: Supplementary file 1 [file oncotarget-12-783-s001.pdf]

Urine protein biomarkers of bladder cancer arising from 16-plex antibody-based screens

SUPPLEMENTARY MATERIALS

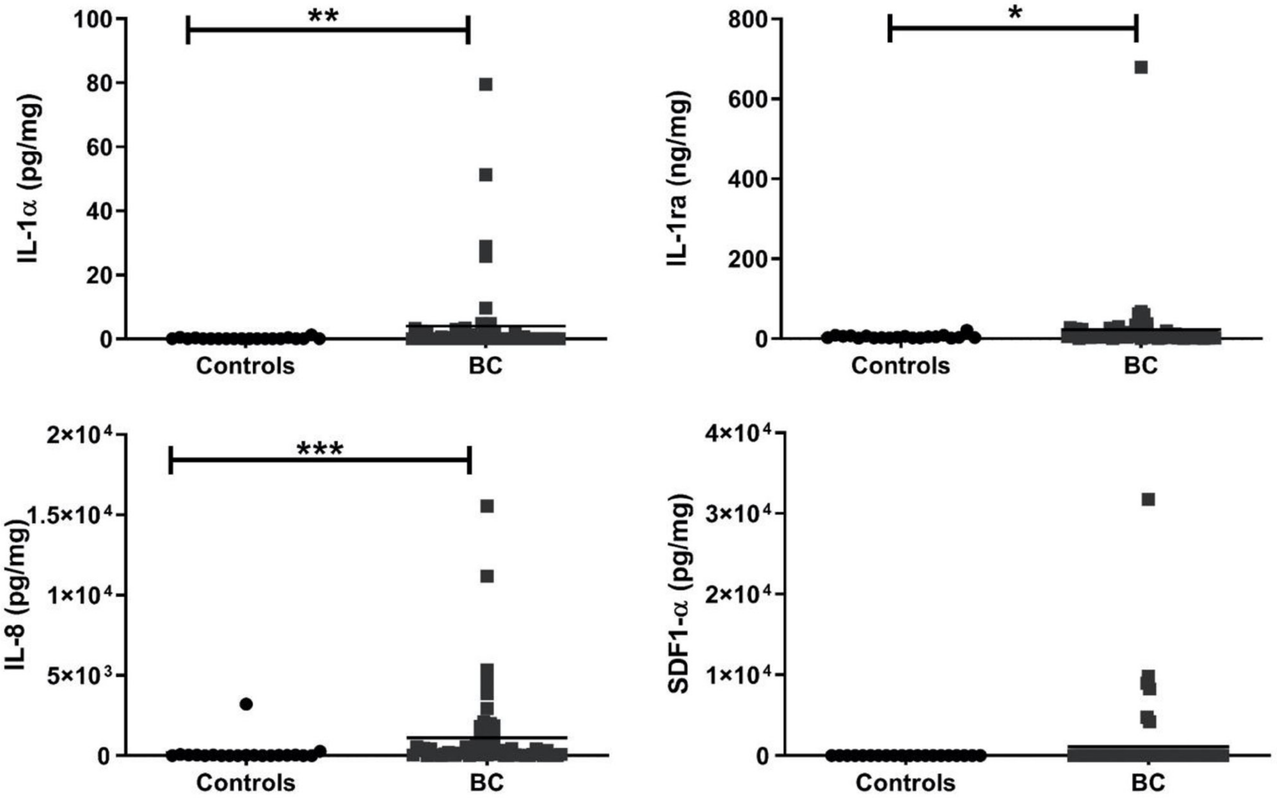

Supplementary Figure 1: ELISA validation of elevated urine proteins in bladder cancer patients. The dot plots depict the 4 proteins selected for ELISA validation based on the initial Luminex screen. Urine from 20 controls and 60 BC patients were tested. Creatinine normalized urine protein levels are indicated as black dots for controls and BC. The asterisks designate the level of significance between controls and BC: $^{*}=p < 0.05$, $^{**}= p < 0.01$, $^{***}=p < 0.001$, and $^{****}=p < 0.0001$, using a chi square test. The corresponding *t*-test values were (IL-1α (0.02), IL-8 (0.02), IL-1ra (0.12), SDF-1α (0.06)). The controls (drawn from Urology clinic) included patients investigated for hematuria but found not to have any urological cancers.

Supplementary Table 1: Urinary protein biomarker concentration in bladder cancer, as validated by ELISA. See Supplementary Table 1

Supplementary Table 2: Sixteen urinary proteins assayed in bladder cancer using Luminex-Based screening. See Supplementary Table 2
