# Supplementary material for: Urine protein biomarkers of bladder cancer arising from 16-plex antibody-based screens
Source: Oncotarget. 2021 Apr 13;12(8):783–90. doi: 10.18632/oncotarget.27941 (PMC8057279; doi:10.18632/oncotarget.27941)
Supplement: Supplementary file 2 [file oncotarget-12-783-s002.docx]

| **Supplementary Table 1: Urinary protein biomarker concentration in bladder cancer, as validated by ELISA** | | | | | | | | | | | | | |
| --- | --- | --- | --- | --- | --- | --- | --- | --- | --- | --- | --- | --- | --- |
| **Creatinine normalized Urinary Protein, pg/mg, Mean (Median)^2^** | | | | | | | **Fold Change^1^** | **Comparison of BC vs. UC^1^** | | | | | |
| **Urine Protein** | **UC** | **Ta** | **Tis** | **T1** | **≥T2** | **BC** | **BC/UC** | **Cut-off** | **ROC AUC** | **Sens.** | **Spec.** | **NPV** | **PPV** |
| IL-1α | 0.12 (0) | 4.10 (0) | 2.31 (0) | 3.53 (0.01) | 4.62 (0.02) | 3.98 (0) | 33.09*** | 0.51 | 0.66** | 0.39 | 0.95 | 0.35 | 0.96 |
| IL-1 ra | 4.75 (3.28) | 32.39 (5.54) | 2.78 (3.77) | 13.71 (6.96) | 8.26 (4.99) | 22.61 (5.31) | 4.76** | 9.68 | 0.61* | 0.32 | 0.95 | 0.32 | 0.95 |
| IL-8 | 195.16(24.08) | 512.55 (42.70) | 512.98 (78.77) | 2931.06 (1136.09) | 1822.13 (835.06) | 1096.97 (181.56) | 5.62**** | 54.07 | 0.77**** | 0.70 | 0.85 | 0.49 | 0.93 |
| SDF-1α | 0 (0) | 0 (0) | 1789.58 (0) | 5090.20 (0) | 1508.02 (0) | 1129.43 (0) | N/A | 4190.79 | 0.54* | 0.09 | 1.00 | 0.27 | 1.00 |
| 1: Indicated are the statistical significance p-values as determined by chi square test (*, P <0.05; **, P <0.01; ***, P <0.001; ****, P<0.0001) | | | | | | | | | |  |  |  |  |
| 2. Independent cohort used for Elisa validation N=80 (20 UC, 35 Ta, 5 Tis, 8 T1, and 12 ≥T2)  3. Sens.=Sensitivity; Spec.=Specificity  The controls (UC, drawn from Urology clinic) included patients investigated for hematuria but found not to have any urological cancers | | | | | |  |  |  |  |  |  |  |  |
