# Supplementary material for: Urine protein biomarkers of bladder cancer arising from 16-plex antibody-based screens
Source: Oncotarget. 2021 Apr 13;12(8):783–90. doi: 10.18632/oncotarget.27941 (PMC8057279; doi:10.18632/oncotarget.27941)
Supplement: Supplementary file 3 [file oncotarget-12-783-s003.docx]

| **Supplementary Table 2: Sixteen urinary proteins assayed in bladder cancer using Luminex-Based screening** | | | | | | | | | | | | | | | | | | |
| --- | --- | --- | --- | --- | --- | --- | --- | --- | --- | --- | --- | --- | --- | --- | --- | --- | --- | --- |
| **Urine Protein, pg/mg, Mean (Median) in different subject groups** | | | | | | | **FoldChange^1^** | | | **Comparison of UC vs. BC^1^** | | | | | | |  |  |
| **Urine Protein** | **UC** | **Ta** | **Tis** | **T1** | **T2-T4** | **UC** | | **BC/UC** | **Cut-off** | | **ROC AUC** | **Sens.** | **Spec.** | **NPV** | **PPV** |  |  |  |
| Eotaxin | 3.86 (2.17) | 60.12 (4.65) | 11.80 (12.24) | 24.56 (10.57) | 45.39 (44.65) | 34.93 (10.98) | | 9.05**** | 6.26 | | 0.81**** | 0.69 | 0.87 | 0.71 | 0.86 |  |  |  |
| GROα | 2.16 (0) | 67.13 (3.16) | 21.76 (0) | 10.24 (3.24) | 108.38 (11.12) | 55.42 (3.16) | | 25.66*** | 1.35 | | 0.71**** | 0.57 | 0.84 | 0.63 | 0.8 |  |  |  |
| IFNα | 0 (0) | 4.05 (0) | 0 (0) | 0 (0) | 0 (0) | 0.81 (0) | | N/A | 28.35 | | 0.51 | 0.03 | 1 | 0.46 | 1 |  |  |  |
| IL-1α | 0.15 (0) | 3.81 (0) | 0.83 (0) | 7.93 (0) | 36.99 (1.01) | 14.41 (0) | | 96.07*** | 0.21 | | 0.70**** | 0.46 | 0.94 | 0.6 | 0.89 |  |  |  |
| IL-1ra | 2813.18 (2483.75) | 30252.58 (15460.44) | 12955.51 (5027.81) | 26719.58 (6134.55) | 17473.15 (17618.86) | 20980.83 (11223.51) | | 7.46**** | 6706.77 | | 0.81**** | 0.63 | 0.94 | 0.69 | 0.92 |  |  |  |
| IL-7 | 2.76 (1.39) | 15.09 (1.84) | 2.34 (1.34) | 7.93 (1.27) | 3.06 (1.93) | 6.39 (1.92) | | 2.32 | 1.92 | | 0.59 | 0.51 | 0.68 | 0.55 | 0.64 |  |  |  |
| IL-8 | 20.05 (5.64) | 171.10 (11.86) | 282.55 (17.76) | 264.93 (49.46) | 654.75 (456.97) | 373.21 (140.13) | | 18.61**** | 28.46 | | 0.81**** | 0.66 | 0.9 | 0.69 | 0.89 |  |  |  |
| IL-15 | 3.08 (0) | 236.98 (0) | 2.82 (0) | 1.09 (0) | 1.62 (0) | 48.88 (0) | | 15.87 | 1.73 | | 0.52 | 0.29 | 0.77 | 0.49 | 0.59 |  |  |  |
| IL-31 | 0 (0) | 142.18 (0) | 15.38 (0) | 1.22 (0) | 2.32 (0) | 33.40 (0) | | N/A | 6.92 | | 0.57* | 0.14 | 1 | 0.51 | 1 |  |  |  |
| IP-10 | 15.32 (4.55) | 134.45 (35.93) | 48.27 (17.64) | 80.63 (30.22) | 116.68 (29.57) | 94.40 (29.57) | | 6.16*** | 15.33 | | 0.77**** | 0.7 | 0.84 | 0.72 | 0.82 |  |  |  |
| MIP-1α | 2.08 (0) | 69.97 (0) | 3.06 (0) | 1.77 (0.51) | 54.06 (1.21) | 32.18 (0) | | 15.47* | 1 | | 0.64* | 0.49 | 0.87 | 0.6 | 0.8 |  |  |  |
| MIP-1β | 4.12 (0) | 130.86 (0) | 22.72 (0) | 22.16 (12.77) | 113.99 (26.02) | 72.91 (12.03) | | 17.70*** | 5.21 | | 0.76**** | 0.68 | 0.84 | 0.7 | 0.82 |  |  |  |
| MCP-1 | 229.01 (208.63) | 493.94 (469.27) | 1152.97 (289.85) | 367.95 (165.06) | 589.62 (100.54) | 664.68 (197.97) | | 2.9 | 676.68 | | 0.52 | 0.29 | 0.97 | 0.54 | 0.91 |  |  |  |
| RANTES | 5.76 (1.19) | 239.95 (6.86) | 16.13 (2.28) | 66.69 (5.00) | 15.11 (7.59) | 72.13 (5.95) | | 12.52** | 2.06 | | 0.69** | 0.77 | 0.65 | 0.71 | 0.71 |  |  |  |
| SDF-1α | 98.93 (78.05) | 673.83 (180.46) | 218.20 (86.15) | 229.81 (127.06) | 411.65 (189.11) | 372.78 (160.22) | | 3.77*** | 139 | | 0.75**** | 0.6 | 0.87 | 0.66 | 0.84 |  |  |  |
| TNFβ | 0 (0) | 0 (0) | 13.55 (0) | 1.61 (0) | 0 (0) | 3.85 (0) | | N/A | 10.64 | | 0.54 | 0.09 | 1 | 0.49 | 1 |  |  |  |
| 1: Indicated are the statistical significance p-values as determined by Mann Whitney U test (*, P <0.05; **, P <0.01; ***, P <0.001; ****, P<0.0001)  The Urology clinic controls (UC) comprised of urology clinic controls, without any urological cancers. | | | | | | | | | | | | | | | | | |  |
